# Physiological Responses of Jurkat Lymphocytes to Simulated Microgravity Conditions

**DOI:** 10.3390/ijms20081892

**Published:** 2019-04-17

**Authors:** Caterina Morabito, Paola Lanuti, Giusy A. Caprara, Marco Marchisio, Mariano Bizzarri, Simone Guarnieri, Maria A. Mariggiò

**Affiliations:** 1 Department of Neuroscience, Imaging and Clinical Sciences, University “G. d’Annunzio” of Chieti-Pescara, 66100 Chieti, Italy; guarnie@unich.it (S.G.); mariggio@unich.it (M.A.M.); 2 Centro Scienze dell’ Invecchiamento e Medicina Traslazionale (CeSI-MeT), University “G. d’Annunzio” of Chieti-Pescara, 66100 Chieti, Italy; paola.lanuti@unich.it (P.L.); marco.marchisio@unich.it (M.M.); 3 Department of Medicine and Aging Sciences, University “G. d’Annunzio” of Chieti-Pescara, 66100 Chieti, Italy; 4 Department of Physiology and Biophysics, University of Colorado Anschutz Medical Campus, Aurora, CO 80045, USA; GIUSY.CAPRARA@UCDENVER.EDU; 5 Department of Experimental Medicine, Sapienza University of Rome, 06100 Rome, Italy; mariano.bizzarri@uniroma1.it

**Keywords:** simulated-microgravity, lymphocytes, oxidative stress, cytoskeletal remodelling, cell shape

## Abstract

The presence of microgravity conditions deeply affects the human body functions at the systemic, organ and cellular levels. This study aimed to investigate the effects induced by simulated-microgravity on non-stimulated Jurkat lymphocytes, an immune cell phenotype considered as a biosensor of the body responses, in order to depict at the cellular level the effects of such a peculiar condition. Jurkat cells were grown at 1 g or on random positioning machine simulating microgravity. On these cells we performed: morphological, cell cycle and proliferation analyses using cytofluorimetric and staining protocols—intracellular Ca^2+^, reactive oxygen species (ROS), mitochondria membrane potential and O_2_^−^ measurements using fluorescent probes—aconitase and mitochondria activity, glucose and lactate content using colorimetric assays. After the first exposure days, the cells showed a more homogeneous roundish shape, an increased proliferation rate, metabolic and detoxifying activity resulted in decreased intracellular Ca^2+^ and ROS. In the late exposure time, the cells adapted to the new environmental condition. Our non-activated proliferating Jurkat cells, even if responsive to altered external forces, adapted to the new environmental condition showing a healthy status. In order to define the cellular mechanism(s) triggered by microgravity, developing standardized experimental approaches and controlled cell culture and simulator conditions is strongly recommended.

## 1. Introduction

The normal functioning of the cells and tissues is influenced by their external environment, including extracellular chemicals and/or physical forces. It is well known that gravity, during the evolution of living matter on Earth has been the main force in shaping of all biological systems [1].

During the last four decades, considering the growing increase of the number of space flights, numerous studies have improved the knowledge on the effects of microgravity on man. The most obvious and rapid alterations induced by microgravity are direct on musculo-skeletal apparatus, neurovestibular, cardiovascular and immune system [2,3,4].

The strong impairment of the immune system, already observed in the astronauts since the first space missions in the 1970s, represents a critical element because this increase the risks of infections or of cancerogenesis. Consequently, further in vitro and in vivo studies investigating how the microgravity influences the immune system are needed. In particular, in vitro observations, under real or simulated microgravity, on isolated lymphocytes have shown that some alterations in molecular mechanisms and signal transduction processes are a direct consequence of gravity modifications [5].

However, as also discussed by Hauschild and colleagues [5], the peculiar response-variability of T cells to external stimuli requires rigorous and standardized experimental cell culture conditions, in order to better compare the results obtained in real and simulated microgravity.

Most experiments with immune cells in simulated microgravity were performed using two-dimensional clinostats, rotating wall vessels (RWVs), random positioning machines (RPMs) [5,6]. Many experiments performed in simulated microgravity conditions, revealed an inhibitory effect of the mitogenic activation process [7,8], and modifications of many cellular features, such as those of cytoskeletal structures, cell cycle, signal transduction, cytokine production, gene and miRNA expression [9,10,11].

These results mainly reflect the immune cell behaviour observed under real microgravity conditions. However, the mechanism by which microgravity alters immune cell functions remains unclear. In lymphocytes, as well as in other cell types, the cytoskeleton plays an important role in maintaining cell shape, to provide mechanical support, to coordinate cell locomotion, to preserve the organelles and cellular proteins in their specific position and it is the main target of external forces [12]. Cytoskeleton alterations induced by internal and external physical forces can affect local mechanical properties and cellular processes, as signal transduction, cell growth and metabolism [13,14]. In addition, many experimental evidences suggest that the cytoskeleton may be a gravity sensor [15,16]. In this scenario, microgravity, influencing immune cell cytoskeleton, may play an important role in immune dysfunction during human permanence in space.

Changes in the organization of vimentin filaments and a reduced patching of the mitogen agent were observed in Jurkat cells exposed to real microgravity on a Maxus rocket flight [17]. In the same cell model, Lewis and colleagues showed that growth responsiveness to microgravity was associated with microtubule anomalies and mediated by apoptosis. Indeed, using DNA microarray analysis, some cytoskeleton-related genes were found up-regulated in Jurkat cells exposed for 48 h to spaceflight microgravity [18,19].

The aim of the present study was to investigate the effects induced by simulated microgravity (s-microgravity) on non-stimulated human T lymphocytes analyzing both cell shape modifications and cell metabolic response, using live cell imaging, in order to correlate these events and to draw a picture of the whole cell response to changes in gravity force. 

A human T lymphoblastoid cell line (Jurkat cells) was chosen as the in vitro model due its easy handling and the known sensitivity to real and simulated microgravity [5]. To simulate a controlled microgravity environment, we used the RPM, largely used and widely accepted as a valid tool for studies on adherent cells and culture suspensions [6,20].

This experimental plan and approach may be useful to improve the knowledge of how the immune cells sense the variations of extracellular gravitational forces and convert them into intracellular signalling.

## 2. Results

### 2.1. Cell Shape Aspects Affected by S-Microgravity

The cell shape, size and complexity were tested after cell exposure to s-microgravity environment produced by the RPM. A recent type of automated cellular imaging technology, that combines the capabilities of microscopy and flow cytometry in a single platform, was used to identify a quantitative cell shape parameter represented by the circularity index [21]. This parameter describes how irregular the membranes of cells are. This is calculated by measuring the radii of the cell from a centroid to the plasma membrane in various sectors of the cell, and is inversely related to the variance of these measured radii. Therefore, a cell with a low radial variance shows high circularity, while a cell with low circularity has a high radial variance and irregular shape. In addition, cell size and complexity were related to flow cytometry analyses using the forward scatter (FSC) parameter that is roughly correlated to the size of the cell, and the side scatter (SSC) parameter, that is an indication of the cell’s granularity, including optical complexity caused by particulate material contained within the cell [22].

These analyses were performed on non-exposed cells (Ctr) and cells exposed to s-microgravity (RPM) up to 96 h, in order to follow the behaviour of at least two cell generations.

**Figure 1:**
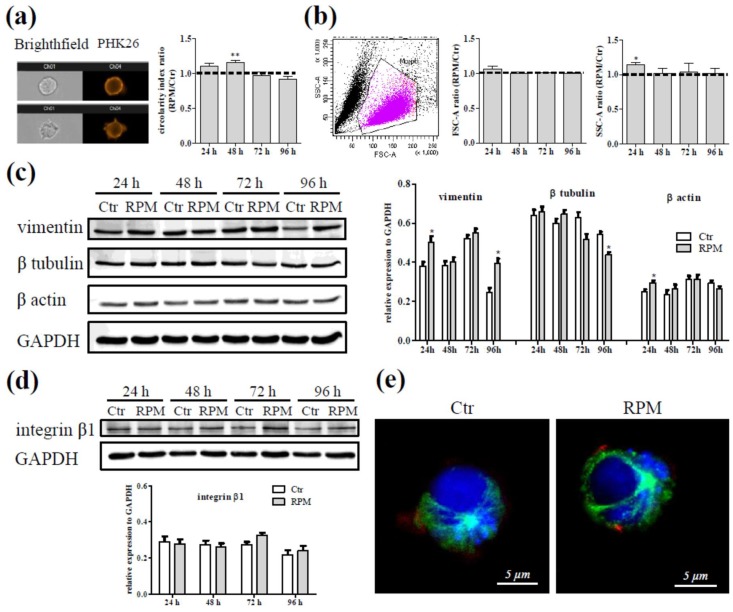
Effect of s-microgravity on cell shape and morphology. (**a**) Representative bright field and fluorescence images of a cell with high (left up) and low (left down) circularity index. The graph on the right shows the ratio between the circularity index of cells exposed to s-microgravity (random positioning machine (RPM)) and that of the corresponding controls (Ctr), for 24, 48, 72 and 96 h. (**b**) A representative forward and side scatter area (forward scatter (FSC)-A and side scatter (SSC)-A respectively) dot-plot, the values from dot-plot analyses of Ctr or RPM-exposed cells at 24, 48, 72 and 96 h, are collected on graphs and reported as the ratio between the mean of FSC-A or SSC-A at RPM conditions and the corresponding controls at each time point. (**c**,**d**) Representative immunoblots of vimentin, β tubulin, β actin and integrin β1 expression levels and densitometric analyses. In particular, the representative blots of β tubulin, β actin and glyceraldehyde-3-phosphate dehydrogenase (GAPDH) (**c**) were cropped from the same membrane, the immunoblot of vimentin (**c**) from a different membrane, that of integrin β1 and GAPDH (**d**) from another membrane. The densitometric analyses are plotted as relative expression calculated by the ratio between OD × mm^2^ of each band and OD × mm^2^ of the corresponding band of the GAPDH, considered as loading control, probed on the same membrane. The relevant proteins were detected using chemiluminescence kits (Pierce EuroClone S.p.A., Pero, Italy) and the signals were acquired and analysed using an image acquisition system (Uvitec mod Alliance9.7, Uvitec, Cambridge, UK), densitometric data were plotted using the Prism5 software (GraphPad, San Diego, CA, USA). In the densitometric analyses, data are means ± SEM from three independent experiments. (**e**) Representative images of Ctr or RPM cells stained with anti-tubulin (green fluorescence), phalloidin-Alexa 594 (red fluorescence) and Draq5 (blue fluorescence). Values of the histograms in a, b, c, and d are presented as means ±SEM, *n* = 3. * *p* < 0.05 compared with the corresponding Ctr.

Our results showed a slight increase of the circularity in Jurkat cells exposed to s-microgravity for 24 h in comparison to the controls. This increase became significant after 48 h of cell exposure. There were no differences after 72 and 96 h between exposed cells and the corresponding controls (Figure 1a). 

The exposure to s-microgravity did not alter the size of the Jurkat cells (Figure 1b, FSC-A), conversely the cell complexity, represented by the values of SSC-A intensity, was greater in 24 h-exposed cells in comparison to control cells, even if longer exposure time did not affect this parameter (Figure 1b, SSC-A). Nevertheless, the D’Agostino & Pearson test assessed a normal data distribution for all samples analyzed, therefore no evidence of different shaped cell sub-populations in the same sample exposed to s-microgravity was detected. 

Cell shape dynamics were strictly related to plasma membrane and cytoskeleton proteins, for these reasons the expression levels and localization of some of these proteins were tested by Western blot and immunofluorescence analyses.

Cytoskeletal proteins showed different expression patterns in exposed cells: a) vimentin expression levels appeared increased after 24 and 96 h of exposure; b) β tubulin appeared reduced after 96 h of exposure and c) β actin appeared increased after 24 h of exposure (Figure 1c). The expression levels of integrin β1 did not change in Jurkat cells at any exposure times (Figure 1d). 

Anyway, the architecture of cytoskeleton in s-microgravity-exposed cells did not appear modified in cells exposed to s-microgravity as shown in the representative images of panel e in Figure 1. These results suggest that s-microgravity could transiently affect cytoskeleton dynamics.

### 2.2. Biological Features in Presence of S-Microgravity

**Figure 2:**
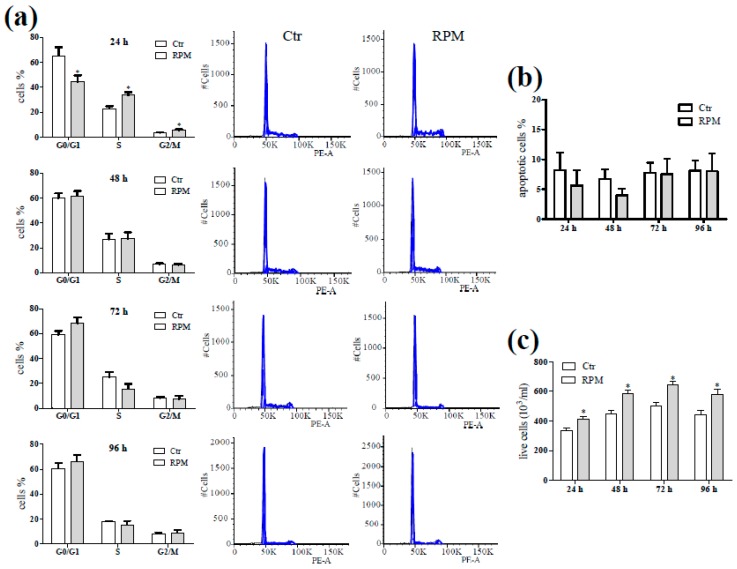
Cell cycle progression and proliferation. (**a**) The percentage of cells in different phases of the cell cycle after exposure under standard (at 1 g, Ctr) or s-microgravity (RPM) conditions, at 24, 48, 72 and 96 h of culturing. The graphs on the sides of the histograms are representative cell cycle profiles of Ctr and RPM cells cultured for 24–96 h. (**b**) Percentages of apoptotic cells derived from cytofluorimetric analysis of the hypodiploid populations. (**c**) Cell growth detected by the trypan exclusion assay on Ctr or RPM conditions. Values of the histograms are presented as means ± SEM, *n* = 3. * *p* < 0.05 compared with the corresponding Ctr.

During the exposure to s-microgravity, cell distribution in the cycle phases was transiently modified after 24 h in comparison to non-exposed control cells. Indeed, only at 24 h of exposure the cell percentages in G0/G1-phase was lower, and those in S- and G2/M-phases were higher than the control ones (Figure 2a). No difference was observed in the percentage of apoptotic cells (evaluated by the analysis of the hypodiploid peaks) that remained under the 15% of cell population (Figure 2b). The exposed cells showed an increased cell number starting from 24 h and an increase of the duplication rate within the 48 h in comparison to control cells (Figure 2c). The Jurkat cells were cultured in growth medium without any external chemical stimulus, in this condition we assayed if the exposure to s-microgravity could trigger an activated status of these cells or the release of interleukins. Cytofluorimetric analyses revealed that both control and s-microgravity exposed cell populations showed a percentage of CD25+ cells lower than 1.0% suggesting the absence of an activated status in any conditions. This result was confirmed by a hardly detectable release of IL-2, TNFα and GM-CSF in the medium in any tested condition (Table S1 in Supplementary Materials).

### 2.3. Intracellular Ca^2+^ Dynamics and Cell Metabolic Features in Response to S-Microgravity Exposure

**Figure 3:**
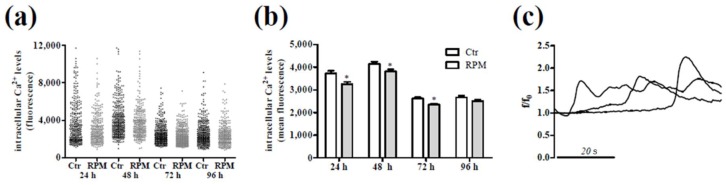
Intracellular Ca^2+^ signalling in single Jurkat cells grown under standard (at 1 g, Ctr) or s-microgravity (RPM) conditions. (**a**) Intracellular Ca^2+^ levels recorded in single cells and expressed as fluorescence intensity. (**b**) The mean basal level of Ca^2+^ expressed as fluorescence mean intensity. Values are presented as means ± SEM. * *p* < 0.05 compared with the corresponding Ctr. (**c**) Representative spontaneous calcium variations that can be recorded in single cells from both Ctr (*n* = at least 312) and RPM exposed (*n* = at least 254) samples.

The analyses of some morphological aspects and biological features were supplemented with a picture of the metabolic state of the cells. To this purpose, we considered intracellular Ca^2+^ levels and oxidative balance. Figure 3 shows data from intracellular Ca^2+^ analyses performed on single cells after growing at 1 g (Ctr) or under s-microgravity conditions (RPM). The quantitative analyses revealed a more homogeneous cell population and lower mean basal levels of intracellular Ca^2+^ starting from 24 h of exposure in comparison to the corresponding controls (Figure 3a,b). In addition, spontaneous intracellular Ca^2+^ variations were observed in both control Jurkat cells as well as in exposed cells (see Figure 3c for representative traces). These results revealed that the spontaneous Ca^2+^ variations and their global kinetics were not related to the growth conditions, at 1× *g* or RPM. On the other hand, the mean amplitude of these oscillations was lower in exposed cells in respect to that observed in control cells, even if the frequency of oscillation and the number of cells showing this spontaneous activity did not differ between control and exposed cells (Table 1).

**Figure 4:**
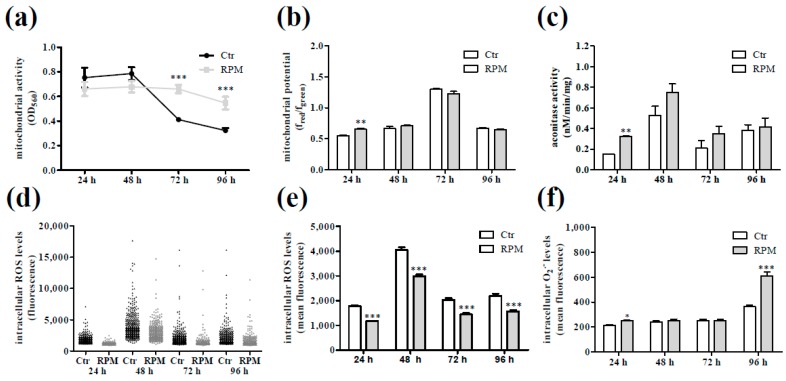
Oxidative status and mitochondria activity of Jurkat cells grown under standard (at 1 g, Ctr) or s-microgravity (RPM) conditions. (**a**) Mitochondria activity tested by 3-(4,5-dimethylthiazol-2-yl)-2,5-diphenyltetrazolium bromide (MTT) assay in live cells, and expressed as optical density (OD) at 560 nm. (**b**) Mitochondria membrane potential in live cells, and expressed as the ratio between red and green fluorescence at each time point. (**c**) Aconitase activity expressed as nmol/min/mg of protein. (**d**) and (**e**) Intracellular ROS levels expressed as fluorescence intensity in single cells (**d**) or mean level of intracellular ROS in cell populations expressed as fluorescence mean intensity (**e**). (**f**) O_2_-levels in cell populations expressed as mean fluorescence intensity. Values in a, b, c, e and f are presented as means ± SEM, *n* = 8. * *p* < 0.05, ** *p* < 0.01 and *** *p* < 0.001 compared with the corresponding Ctr.

The exposed cells showed also a metabolic status different from control cells, as revealed by the trend of the mitochondria activity along the growth period (Figure 4a). This was accompanied with a higher mitochondria membrane potential and aconitase activity at 24 h-exposed cells in comparison to control cells at the same growth time (Figure 4b,c). In addition, fluorescence analyses showed a lower intracellular reactive oxygen species (ROS) levels at every time (Figure 4d,e) with a higher mitochondria superoxide anion production in 24 h- and 96 h-exposed samples (Figure 4f).

**Figure 5:**
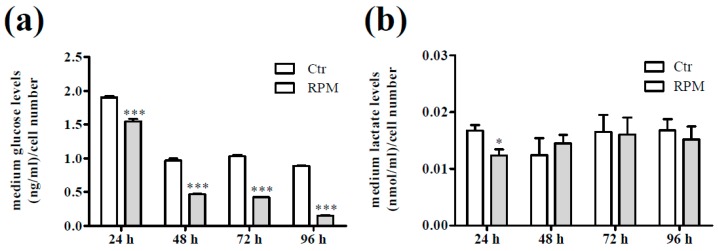
Cellular metabolism after exposure to s-microgravity. (**a**) Glucose levels were measured in the growth medium of cells grown at 1 g (Ctr) or exposed to s-microgravity (RPM) and are expressed as the ratio between glucose levels (ng/mL) and the number of cells at each time point. (**b**) Lactate levels were measured in cell medium from cells grown under the same conditions as described in (**a**) and are expressed as the ratio between lactate levels (nmol/mL) and the number of cells at each time point. Values are presented as means ± SEM, *n* = 3. * *p* < 0.05, *** *p* < 0.001 compared with the corresponding Ctr.

The content of glucose was lower in the medium from every time exposed cells and the content of lactate was lower only in the medium from 24 h-exposed cells in comparison with the corresponding controls (Figure 5a,b). 

## 3. Discussion

The negative impact of space flights on immune system was revealed since the first attempts to navigate and colonize the space by direct (such as an immune cell dysfunction in astronauts’ blood sample) or indirect (such as the increased risk of bacterial and viral infections) evidences [23,24,25]. The astronauts themselves are a sort of “live incubator” for immune cells, so that only drawing blood samples is possible to isolate cells that were exposed to space environment, also facilitate the study of the immune cell response to space flights. On the other hand, it has to be considered that during space exposure, the live matter was not only exposed to microgravity but also to atmospheric dust, radiation and temperature oscillation, above all during extravehicular walks. All these conditions affect, or may affect at different extent, the immune system functions [24,26]. To define the mechanism(s) induced by each space environment stressor on immune cells in vitro models and simulators are necessary also to determine countermeasure strategies to protect human health.

Under this perspective, some bioreactors that simulate microgravity have been designed and built [6]. In this study, we used the RPM that for its physical and technical features is a well-recognized and accepted microgravity simulator [20]. Indeed, its size allowed its positioning inside a cell culture incubator and it is driven by a dedicated software that checks its correct functioning in order to perform reproducible and standardized experiments. Using the RPM-generated s-microgravity, we exposed Jurkat lymphocyte cells with the aim of mirroring the response of human lymphocytes to a such peculiar stimulus. As previously reported also by other authors [15,27,28], control and exposed cell cultures were maintained in the same conditions in T25-flaks completely filled with culture medium to avoid effects by buoyancy and shear stress during rotation. In any case, we cannot completely exclude a possible side effect induced by these forces, and we think that it is an intrinsic limit of on-ground microgravity-simulating devices. In our knowledge, it is hard to calculate or to generate a theoretical model to define possible disturbing mechanical stimuli during the functioning of microgravity simulators. This is due to the so many differences in cell models (adherent cells, cell suspension, tissue-like cell organization, as well as cell size, cell interactions, cell density) and their containers (shape and size). 

Recently, Hauslage and colleagues [29] demonstrated that a biological response was dependent on the device settings. Using a cell culture of *Pyrocystis noctiluca* as a biosensor of mechanical force variations, the Authors reported that a side effect induced by shear forces was present when the cells were exposed to RPM in the random mode running, in comparison to the presence of negligible shear forces during a one-axis clinorotation exposure. These results were obtained using small volume cuvettes, and the Authors concluded that further experiments should be performed using larger volumes [29].

In addition, Wuest and colleagues [30], using a numerical approach, defined the fluid dynamic characteristic occurring inside a cell culture flask turning on an operating RPM, a condition mirroring that of the present study. Their simulations showed that the possible highest shear stresses are found along the flask walls (up to a few 100 mPa), and the shear stresses in the inner space of the flask are smaller, and their magnitude is in the order of 10 mPa, a suitable condition for cell in suspension as the Jurkat cells are.

Our choice of this cellular model was based on the consideration that a cell line is a stable cellular model easily to be managed and lymphocytes can be considered as biosensors of the whole-body response and adaptation to environmental conditions [31,32,33,34,35]. After the exposure, the cell morphology was analyzed on live cells using an automated cellular imaging technology, the Amnis that combining the microscopy and flow cytometry in a single platform, is able to perform morphological analysis and calculation of quantitative indices.

The modification of external forces caused by s-microgravity conditions induced transient modifications in cell shape and intracellular density in Jurkat cells, accompanied by transient changes in the expression levels of some cytoskeletal proteins such as actin, tubulin and vimentin that are not only mere structural proteins but also the main functional milestones in the cytoskeleton dynamics [36,37,38].

Notably, in these Jurkat cells exposed to s-microgravity, the presence of co-existent cell sub-populations characterized by different discrete forms were not detected, differently from other cell phenotypes featured by cell-cell interactions [39]. We can speculate that, the shape constraints driven by gravity are also related to a tissue-like organization with relevant cell-cell interactions. This feature is not present in lymphocytes that are isolated cells circulating in a fluid environment. 

The transiency of this effect on cell shape and the cell adaptation to s-microgravity is supported by the evidence that the global cytoskeleton organization was not affected and also the exposed cells showed a microtubule organizing center from which microfilaments radiated. This allowed the cells to correctly duplicate even at a higher rate during the first 24 h-exposure without showing relevant apoptotic features. After the first 24 h-burst the duplication rates of control and exposed cells were similar during the following days of culture.

These results did not fully agree with those previously published. Indeed, there are numerous studies regarding the effects of microgravity on immune T cells based on a large variability in cell models and environmental conditions [5]. As reviewed by Hauschild and collaborators [5], there are numerous variables in the experimental plan even selecting the studies regarding only Jurkat cells (such as: different media composition, exposure to real or simulated microgravity, time of exposure, etc.). In addition, cell exposure to space-environment could represent an environment “contaminated” by other stimuli (such as cosmic radiation) beyond the microgravity. Most of these studies referred an altered cell proliferation, induction of apoptosis and cytoskeleton disruption on flown cells, all features that could be influenced also by other stimuli present in space [40,41]. In addition, for each flight or space mission a limited amount of experiments can be performed mainly due to the small dimension of cell vessel and to the high costs, these aspects do not allow to compose a holistic puzzle of the behaviour of the cell in conditions of microgravity.

Our experimental plan was designed in order to continuously expose non-stimulated Jurkat cells, a human T cell model maintained in 10% serum-supplemented medium to preserve a proliferative status, up to a 96 h period compatible to a long lasting exposure. Our in vitro microgravity simulator is not equipped with accessories that allow biological tests during the exposure or soon after short-time exposures. On the other hand, the increased number of cells and depleted medium that cannot be renewed would affect exposures longer than 96 h.

In our experimental conditions, the transitory effects induced by s-microgravity on cell shape and proliferation were accompanied by metabolic changes above all related to mitochondrial activity. 

Monitoring intracellular Ca^2+^ levels, the exposed cells, compared to control ones, appeared in a more homogeneous and rather quite status showing lower basal Ca^2+^ and spontaneous ionic oscillations with lower amplitude. These data revealed an improbable cell activation that, conversely, is often related to an increased intracellular Ca^2+^ mobilization in Jurkat cells [42,43]. In addition, the very low CD25+ cell percentage and low amount of IL2 in the medium remove any doubt about a possible activation status of lymphocytes during exposure. This aspect was in accordance with the slight and transient modifications in the cytoskeleton organization whose dynamics are bi-directionally linked to intracellular Ca^2+^ homeostasis [44,45]. This scenario was completed by the analyses of the activity of mitochondria that are not only important for energy supply but have other important cellular functions including Ca^2+^ buffering and T cell activation [46]. During Jurkat cells exposure to s-microgravity, we observed an increasing mitochondrial activity, conversely to that which happened in control cells. In s-microgravity, mitochondria increased their aerobic activity metabolizing glucose without increasing the production of lactate, increasing the production of oxygen anion that was efficiently detoxified also by the aconitase activity that prevented the possible increase of intracellular ROS levels, thus maintaining a healthy status of mitochondrial membranes. In this scenario, a discordant note outside the chorus, appears with the increased mitochondrial oxygen anion after 96 h of exposure. This could be the first possible sign of cell metabolic requirement considering a decreased glucose level in the medium, and that no renewal of medium was performed while the number of cells in exposed flasks was higher than that in flasks at static 1× *g*.

All these results suggest that non-activated proliferating Jurkat cells after the first 24 h-exposure to s-microgravity adapted to the new environmental condition showing a healthy status. This rapid adaptation is in accordance with results shown by Thiel and colleagues [11] that investigated the gene expression profile in non-activated Jurkat cells in different real and simulated gravity conditions. In their study, the authors concluded that Jurkat cells showed an overall high stability of gene expression in microgravity and hypothesized that the altered transcripts are mostly involved in fast cellular adaptation processes [11]. Moreover, the adaptive capacity is not only confined to the lymphocyte phenotype but it was also described in other cells of the immune system. Tauber and colleagues [47] reported that primary human macrophages did not exhibit quantitative or structural changes of the actin and vimentin cytoskeleton after 11 days in microgravity conditions when compared to 1 g controls, even if some metabolites in the medium resulted significantly changed. These authors assumed that the non-significant cytoskeletal modifications could represent an adaptive state after the long 11-day exposure to microgravity [47].

In conclusion, we strongly agree with Hauschild and collaborators [5], who required to develop experiments with rigorous standardized cell culture and simulator conditions, in order to make it possible to compare results obtained in different laboratories and to depict cellular mechanism(s) triggered by microgravity. In addition, it is important to stress a different inference of microgravity triggered-constraints on tissue-like cell organization or single circulating cell population due to their specific physical features [48].

This, in turn, could open the way and focus the research on developing appropriate protection strategies and countermeasures for long-term missions.

In our experimental conditions, the Jurkat cells found a suitable condition when moved by RPM to simulate microgravity, just as they are continuously moving in the circulatory system. But, in this last condition they are not only exposed to external driving forces but also to chemical stimuli (antigens, growth factors, hormones, etc.). All these physico-chemical factors were modified in the circulatory system in astronauts during space flights so that, even though with appropriate simplifications, such a microenvironment should be recreated in vitro in order to define the physiological responses of lymphocytes to microgravity.

## 4. Materials and Methods

### 4.1. Equipment and Cell Exposure Parameters

A desktop RPM connected to a control console through standard electrical cables (Dutch Space, Leiden, The Netherlands) was used to simulate microgravity conditions. This apparatus is a 3D clinostat consisting of two independently rotating frames, positioned one inside the other. The random mode running exerts a very complex net change in orientation on a biological sample positioned in the centre of the RPM. The angular speed and the inclination of the disk determine the degree of s-microgravity. This device does not actually eliminate gravity, but it is a simulator of microgravity conditions based on the principle of “gravity-vector averaging”. During random mode running, the RPM allows a 1× *g* stimulus to be applied omnidirectionally rather than unidirectionally, consequently the sum of the gravitational force vectors tends to equal zero. The effects of s-microgravity generated by the RPM are comparable to those induced by real microgravity, provided that the direction changes are faster than the time occurred to the system to react to the gravity field. The desktop RPM was sited within an incubator (to maintain the 37 °C temperature, 5% CO_2_ and 95% humidity), it operated in 3D random mode and a mean angular speed of +/−60°/s for both inner and outer frames was applied during sample exposition. These settings allowed to the control console to provide randomly selected and varied rotation directions and this resulted in an unpredictable and symmetrical path [49]. To minimize the effect of possible accelerations due to rotation, the sample was placed in the centre of the RPM [49].

### 4.2. Chemicals and Materials

Unless indicated otherwise, the cell culture medium, sera, and antibiotics were purchased from ThermoFisher Scientific (Monza, Italy), the cell culture plastic ware was obtained from Becton Dickinson Falcon (Steroglass S.r.l., San Martino in Campo, Italy), and the reagents and standards were obtained from Sigma-Aldrich (Milan, Italy).

### 4.3. Cell Cultures

**Figure 6:**
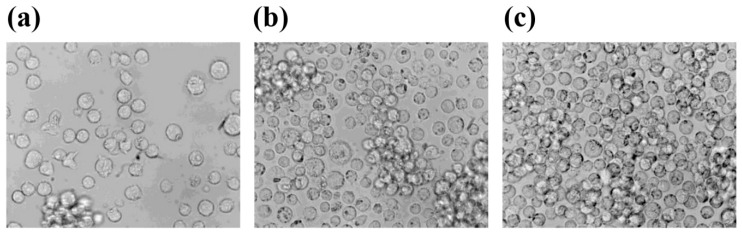
Bright field images of Jurkat cells. he images were acquired using a Leika DMIR microscope with a 40× objective, and show cells soon after cell seeding (**a**) or after 96 h at 1× *g* (**b**) or at RPM (**c**).

The human Jurkat cell line (clone E6.1, ECACC, Salisbury, UK) was cultured in growth medium (RPMI 1640 supplemented with 10% foetal bovine serum and penicillin/streptomycin) at a density of 200 × 10^3^ cells/mL. Experiments were performed on cells cultured up to 96 h at 1 g in the same incubator containing the RPM or at simulated microgravity (s-microgravity) in the RPM in T25 flasks completely filled with culture medium to avoid air bubbles and to minimize liquid flow; thus, the effects of both buoyancy and shear stress during rotation were negligible. The images reported in Figure 6 show the cell appearance in flasks after cell seeding (Figure 6a) or after 96 h at 1 g (Figure 6b) or at RPM (Figure 6c), before cell processing for the experimental analyses.

### 4.4. Viability and Proliferation Assays

The cell viability was tested using the trypan blue exclusion assay. The cells were stained with a trypan blue dye solution (0.5% in phosphate-buffered saline, PBS), and counted using a Bürker chamber. The blue-stained cells were considered non-viable.

### 4.5. Flow Cytometry and Cell Cycle Analysis

Cell cycle investigations were performed according to published protocols [50]. Cell samples from the T25 flasks were fixed with 70% (*v*/*v*) ethanol, washed with PBS and centrifuged (400 × *g*, 10 min at room temperature, RT). Cell pellets were re-suspended and stained with a propidium iodide solution (50 µg/mL propidium iodide and 100 µg/mL RNase in PBS, Sigma-Aldrich, Milan, Italy). The fluorescence intensity was recorded using a FACSCanto II flow cytometer (Becton Dickinson Biosciences, Franklin Lakes, NJ, USA). Samples were cultured in triplicate, and a minimum of 10 × 10^3^ events were acquired for each sample. Aggregates were excluded [51], and the percentages of cells in G0/G1, S, and G2/M phases of the cell cycle, as well as apoptotic and necrotic populations, were calculated using FlowJo software v8.8.6 (TreeStar, Ashland, OR, USA); in particular, the analysis of the cell cycle was obtained applying the Watson Model.

To identify the activated compartment, Jurkat cells (500 × 10^3^/sample), after centrifugation at 400× *g* for 10 min, were washed in PBS and stained using the PE-conjugated anti-CD25 (BD Biosciences, Franklin Lakes, NJ, USA). After a 30-min incubation at 4 °C with the antibody, the cells were washed, re-suspended in 0.5 mL PBS, and 100 × 10^3^ events/sample were acquired using a flow cytometer (FACSCanto II; BD Biosciences, Franklin Lakes, NJ, USA) [52]. The gate of CD25+ cells was placed on the basis of the relative unstained sample. A forward scatter area (FSC-A) vs side scatter area (SSC-A) dot plot was used to identify and gate Jurkat cells. The Cytometer Setup and Tracking Module (BD Biosciences, Franklin Lakes, NJ, USA) was used to test instrument performances and data reproducibility, these were further validated by the acquisition of Rainbow Beads (BD Biosciences, Franklin Lakes, NJ, USA) and compensation was calculated using Comp Beads (BD Biosciences, Franklin Lakes, NJ, USA). Before each analysis, the flow cytometer was cleaned according to the manufacturer instructions, and carryover between samples was prevented using the FACSCanto SIT Flush device. The FACSDiva v6.1.3 and FACSuite v1.05 software (BD Biosciences, Franklin Lakes, NJ, USA) were used to analyse the data after the acquisitions).

### 4.6. Cytokine Levels

The levels of the cytokines, present in the cell-conditioned growth medium, were quantified by a conventional sandwich-based enzyme-linked immunosorbent assay (ELISA) technique using a Multi-Analyte ELISArray kit (Qiagen, Milan, Italy) according to manufacturer’s instructions.

### 4.7. Immunofluorescent Cytoskeleton Detection

The Jurkat cells (500 × 10^3^ cells/sample), were fixed with a 4% paraformaldehyde solution in PBS (15 min), centrifuged (400× *g*, 10 min at RT), washed in PBS, permeabilized with a 0.1% TritonX-100 solution (10 min) and incubated in blocking buffer (PBS plus 10% goat serum) for 1 h at RT. After a 2 h-incubation at RT with the primary antibody (mouse anti-β tubulin,1:50 dilution, Santa Cruz Biotechnology, Heidelberg, Germany), followed by a 1 h-incubation at RT with the secondary antibody (goat anti mouse Alexa Fluor -488, 1:200 dilution, ThermoFisher Scientific, Monza, Italy), the cells were stained with Alexa Fluor 546 Phalloidin (1:50 dilution, ThermoFisher Scientific, Monza, Italy) for 1 h at RT, and the nuclei were counterstained with DRAQ5 (10 µM, ThermoFisher Scientific, Monza, Italy) for 10 min at 37 °C. After a washing with PBS the cells were re-suspended in PBS and plated on poly-l-lysine-coated 12 mm glass coverslips. Images were acquired using a Zeiss LSM5 Pascal confocal microscope (Jena, Germany).

### 4.8. ImageStream Analysis of Cell Shape

For imaging flow cytometry, sample acquisitions were carried out using ImageStream (Amnis, Seattle, WA, USA; one laser, six-color configuration). Cells (5 × 10^3^ cells/sample), labelled with 4 µM PKH26, a red fluorescent membrane probe (Sigma-Aldrich, Milan, Italy), were analyzed by IDEAS software, v. 5.0 (Amnis), The cell shape was analyzed by measuring the “circularity feature” on the fluorescent channel, as previously described [21]. The circularity parameter has been calculated for each cell by the program, as the averaged radius divided by the radial variance. Typical round cells exhibit low radial variance, resulting in high circularity parameter values. Therefore, this feature measures cell relative degree of deviation from a circle, transforming its morphological characteristics in a measurable parameter.

### 4.9. Western Blotting

Cells were collected, sonicated in a cold buffer (50 mM Tris-HCl, pH 7.4, 100 mM NaCl, 50 mM NaF, 40 mM β-glycerophosphate, 5 mM EDTA, 1% Triton X-100, 200 µM sodium orthovanadate, 100 µg/mL PMSF, 10 µg/mL leupeptin, 5 µg/mL pepstatin A, and 10 µg/mL benzamidine), centrifuged (10,000× *g* for 10 min at 4 °C) and supernatants containing cytosolic proteins were recovered. Protein concentrations were determined using a protein assay kit (Bio-Rad DC; Bio-Rad, Segrate, Italy). Cell extracts (40 μg of protein/lane) were separated on 8% or 10% (*w*/*v*) homogeneous SDS-PAGE slab gels and then transferred to nitrocellulose membranes (Protran; Whatman-GE Healthcare, Milan, Italy). Membranes were hybridized with a rabbit polyclonal anti-Integrin β1 (1:500 dilution, Santa Cruz Biotechnology, Heidelberg, Germany), mouse monoclonal anti-β tubulin or β actin antibody (1:1000 dilution, Santa Cruz Biotechnology, Heidelberg, Germany), mouse monoclonal anti-vimentin (1:500 dilution, ThermoFisher Scientific, Monza, Italy) followed by an incubation with horseradish-peroxidase-conjugated anti-mouse or anti-rabbit IgGs (1:10,000 dilution, GE Healthcare, Cologno Monzese, Italy). The relevant proteins were detected using chemiluminescence kits (GE Healthcare), and the signals were acquired and analysed using an image acquisition system (Uvitec mod Alliance 9.7, Uvitec, Cambridge, UK). A mouse monoclonal anti-glyceraldehyde-3-phosphate dehydrogenase (GAPDH) antibody (1:10,000 dilution, Merck S.p.a., Vimodrone, Italy) was used as a loading control. The choice of GAPDH as internal standard was due to the experimental evidences (from our and other laboratories [11]) showing that the expression levels of this protein did not change in any condition we tested (control or s-microgravity exposure at different times).

### 4.10. Measurements of Glucose and Lactate Levels in Cell Culture Medium

Glucose and lactate levels in the growth medium were tested according to the manufacturer instructions, using a Free Style Optium glucometer (Abbot Laboratories, Rome, Italy) and a Lactate Pro Analyser (Arkray Inc. Kyoto, Japan), respectively [28].

### 4.11. Fluorescence Analyses on Single Cell

Intracellular Ca^2+^ and ROS levels were monitored in Jurkat cells using the Fluo4-acetoxymethylester dye (Fluo4/AM, ThermoFisher Scientific, Monza, Italy) or H_2_-DCFDA (ThermoFisher Scientific, Monza, Italy) respectively, and an upright microscope (Zeiss Axio Examiner, Jena, Germany), equipped with a 40× 0.75NA water-immersion objectives. The microscope is connected by an optical fiber to a 75W Xenon lamp and a monochromator (OptoScan; Cairn Instrument, UK). Cells (200 × 10^3^ cells/mL) were incubated in a normal external solution (NES: 140 mM NaCl, 2.8 mM KCl, 2 mM CaCl_2_, 2 mM MgCl_2_, 10 mM glucose and 10 mM HEPES, pH 7.3) supplemented with 10 mg/mL bovine serum albumin and 5 µM of Fluo4-AM or 10 µM of H_2_-DCFDA (Table 1), for 40 min at 37 °C. After a washing, the cells were re-suspended in NES and seeded on poly-l-lysine–coated 35 mm plates at a density of 200 × 10^3^ cells/cm^2^, and maintained for 10 min at RT to allow cell adhesion. The fluorescence images were acquired at 2 frames/s with a 16 bit digital EM-CCD camera (PhotoEvolve 512; Photometrics; Tucson, AZ, USA). For spontaneous Ca^2+^ oscillation analysis, the mean fluorescence intensity signal in a selected cell area was calculated as f/f_0_, where f is the fluorescence intensity of a single loaded cell that was acquired during the time lapse, and f_0_ is the mean fluorescence intensity of the same cell calculated from images acquired during the first 5 s. The oscillations’ amplitude was calculated as the ratio between the maximum peak and the baseline and the frequency as number of peaks per minute [53].

### 4.12. Spectrofluorimetric Measurements

Cells (400 × 10^3^ cells/mL) were incubated with NES containing one of the probes (ThermoFisher Scientific, Monza, Italy) reported in Table 2 for 40 min at 37 °C. After a washing, the cell were re-suspended in NES and seeded on poly-l-lysine–coated special-optic 96-well plates (Corning-Costar, Milan, Italy) at a density of 50 × 10^3^ cells/well, and maintained for 10 min at RT to allow cell adhesion. Then, the samples were rinsed and fluorescence intensity was detected using a microplate reader (Synergy H1 multimode, Biotek, Bad Friedrichshall, Germany), recorded from each well every 30 s for 5 min at 25 °C and stored on a computer using the software GEN5™ (Biotek, Bad Friedrichshall, Germany). The fluorescence values of Fluo-4-, H_2_-DCFDA- or MitoSox RED-loaded cells were expressed as the means (±standard errors of the means, SEM) of the fluorescence value recorded during 5 min of acquisition. Fluorescence values of JC1-loaded cells are expressed as the means (±SEM) of the red/green fluorescence ratio, which depends on the mitochondrial membrane potential. Table 2 collects the probes used in this study and their specific features. For each sample, three independent experiments were performed, each containing eight repetitions [28].

### 4.13. MTT Assay

Metabolic activity was tested using a colorimetric assay based on reduction of 3-(4,5-dimethylthiazol-2-yl)-2,5-diphenyltetrazolium bromide (MTT). At each experimental time, cell samples were withdrawn from T25 flasks of control and s-microgravity exposed cells. After cell counting, cell aliquots were plated at same density (50 × 10^3^ cells/well) on poly-l-lysine-coated 96-well plates and maintained for 10 min at RT to allow cells adhesion. Then, 20 µL MTT solution (5 mg/mL in PBS) was added into each well, and the cells were incubated at 37 °C for 3 h. After a 10-min centrifugation at 500× *g*, the supernatant was removed and discarded, and 200 µL dimethylsulfoxide were added into each well. After a 30 min-incubation at 37 °C, the absorbance was measured at 560 nm with a microplate reader (Synergy H1 multimode, Biotek, Bad Friedrichshall, Germany). For each experimental condition, eight repetitions were performed in three independent experiments [52].

### 4.14. Aconitase Assay

The activity of the enzyme aconitase was performed using an aconitase assay kit (Cayman Chemical, Ann Arbor, MI, USA). Samples, obtained from cells sonicated in cold assay buffer, centrifuged (20,000× *g*, 10 min at 4 °C) and re-suspended in cold assay buffer, were assayed as described by the manufacturer (Cayman Chemical). This assay measures the absorbance of NAD(P)H at 340 nm, which is generated in the coupled reactions of aconitase with isocitric dehydrogenase (enzyme of Krebs cycle). The rate at which NAD(P)H is generated is proportional to the activity of aconitase. Oxidative stress reduces aconitase activity [54].

### 4.15. Statistical Analyses

Experimental data are reported as means ±SEM. Statistical significance was calculated by Student’s *t*-test (Prism5 software, GraphPad, San Diego, CA, USA). *p* values < 0.05 were considered statistically significant. Data were analysed by the D’Agostino & Pearson omnibus normality test to assess data distribution (Prism5 software, GraphPad).

## Figures and Tables

**Table 1:** Quantitative analyses of the spontaneous intracellular Ca^2+^ variations.

Time	Oscillating Cells (% ± SEM)		Frequency of Ca^2+^ Oscillations (Peaks/min ± SEM)		Amplitude of Ca^2+^ Oscillations (Ratio Peak_max_/Baseline)		Tested Cells (Number)	
	Ctr	RPM	Ctr	RPM	Ctr	RPM	Ctr	RPM
**24 h**	49.3 ± 13.7	49.3 ± 9.4	1.06 ± 0.02	1.03 ± 0.02	1.83 ± 0.07	1.45 ± 0.04 ***	312	254
**48 h**	43.0 ± 8.0	41.3 ± 3.3	1.06 ± 0.02	1.05 ± 0.02	1.40 ± 0.03	1.28 ± 0.02 **	364	277
**72 h**	55.3 ± 6.1	41.3 ± 7.5	1.15 ± 0.03	1.15 ± 0.03	1.31 ± 0.02	1.25 ± 0.02 *	357	406
**96 h**	53.1 ± 5.5	55.3 ± 7.7	1.12 ± 0.01	1.12 ± 0.03	1.29 ± 0.02	1.25 ± 0.02	346	294

In the table are reported the percentages of oscillating cells for each experimental condition, frequency of intracellular Ca^2+^ oscillations (reported as the number of peaks per min) and amplitude of the oscillations (calculated as the ratio between the maximum peak and the baseline). Values are presented as means ± SEM. * *p* < 0.05, ** *p* < 0.01, *** *p* < 0.001 compared with the corresponding Ctr.

**Table 2:** Fluorescence probes used for intracellular analyses.

Probe	Excitation (nm)	Emission (nm)	Analyses
Fluo4-AM 5 µM	488	520	Intracellular Ca^2+^ levels
H_2_-DCFDA 10 µM	488	520	Intracellular ROS levels
MitoSox RED 5 µM	510	580	Mitochondrial O_2_^−^ levels
JC1 5 µg/mL	488	520/590	Mitochondrial membrane potential

## Supplementary Materials

Supplementary materials can be found at https://www.mdpi.com/1422-0067/20/8/1892/s1.
