# Supplementary material for: Physiological Responses of Jurkat Lymphocytes to Simulated Microgravity Conditions
Source: Int J Mol Sci. 2019 Apr 17;20(8):1892. doi: 10.3390/ijms20081892 (PMC6515345; doi:10.3390/ijms20081892)
Supplement: Supplementary file 1 [file ijms-20-01892-s001.pdf]

Table 1s. Quantitative analyses of the spontaneous intracellular

Time	IL-2 (OD$_{450}$ ± SEM, n=3)		TNFα (OD$_{450}$ ± SEM, n=3)		GM-CSF (OD$_{450}$ ± SEM, n=3)	
	**Ctr**	**RPM**	**Ctr**	**RPM**	**Ctr**	**RPM**
**24 h**	0.174 ± 0.010	0.155 ± 0.008	0.006 ± 0.00001	0.004 ± 0.000001	0.0005 ± 0.000009	0.0007 ± 0.000007
**48 h**	0.170 ± 0.010	0.158 ± 0.025	0.004 ± 0.00001	0.003 ± 0.000001	0.0005 ± 0.000008	0.0006 ± 0.000008
**72 h**	0.169 ± 0.003	0.148 ± 0.004	0.005 ± 0.00001	0.004 ± 0.000001	0.0004 ± 0.000008	0.0006 ± 0.000009
**96 h**	0.542 ± 0.025	0.433 ± 0.020	0.004 ± 0.00001	0.003 ± 0.000001	0.0005 ± 0.000017	0.0003 ± 0.000023

In the table are reported the levels of interleukins expressed as the mean OD acquired at 450 nm (± SEM) of 3 independent experiments. The levels of the cytokines, present in the cell-conditioned growth medium, were quantified by a conventional sandwich-based enzyme-linked immunosorbent assay (ELISA) technique using a Multi-Analyte ELISArray kit (Qiagen, Milan, Italy) according to manufacturer's instructions. The very low OD values show a hardly detectable levels of these factors in the medium and a non-activation state of the cells.
